# How high resolution 3-dimensional imaging changes our understanding of postnatal lung development

**DOI:** 10.1007/s00418-018-1749-7

**Published:** 2018-11-02

**Authors:** Johannes C. Schittny

**Affiliations:** Institute of Anatomy, University of Bern, Baltzerstrasse 2, 3012 Bern, Switzerland

**Keywords:** Lung development, Pulmonary alveolarization, Microvascular maturation, Angiogenesis, Pulmonary acinus

## Abstract

During the last 10 + years biologically and clinically significant questions about postnatal lung development could be answered due to the application of modern cutting-edge microscopic and quantitative histological techniques. These are in particular synchrotron radiation based X-ray tomographic microscopy (SRXTM), but also ^3^Helium Magnetic Resonance Imaging, as well as the stereological estimation of the number of alveoli and the length of the free septal edge. First, the most important new finding may be the following: alveolarization of the lung does not cease after the maturation of the alveolar microvasculature but continues until young adulthood and, even more important, maybe reactivated lifelong if needed to rescue structural damages of the lungs. Second, the pulmonary acinus represents the functional unit of the lung. Because the borders of the acini could not be detected in classical histological sections, any investigation of the acini requires 3-dimensional (imaging) methods. Based on SRXTM it was shown that in rat lungs the number of acini stays constant, meaning that their volume increases by a factor of ~ 11 after birth. The latter is very important for acinar ventilation and particle deposition.

**Electronic supplementary material:**

The online version of this article (10.1007/s00418-018-1749-7) contains supplementary material, which is available to authorized users.

## Introduction

As part of the celebration of the 60th Anniversary of ‘Histochemistry and Cell Biology’ this review would like to elucidate the contribution of methodological innovations which significantly improved our understanding of postnatal lung development—in particular of classical and continued alveolarization, as well as of the development of the acini. In this context, high resolution synchrotron radiation X-ray tomographic microscopy (Stampanoni et al. 2010; Schittny et al. 2008; Vasilescu et al. 2012; Lovric et al. 2013) represents the most important innovation, but stereological estimation of the number of alveoli (Hyde et al. 2004) and the length of the free septal edge (Schittny et al. 2008), as well as ^3^Helium Magnetic Resonance Imaging (Woods et al. 2006; Yablonskiy et al. 2009) were also contributing.

## Overview of lung development

**Fig. 1:**
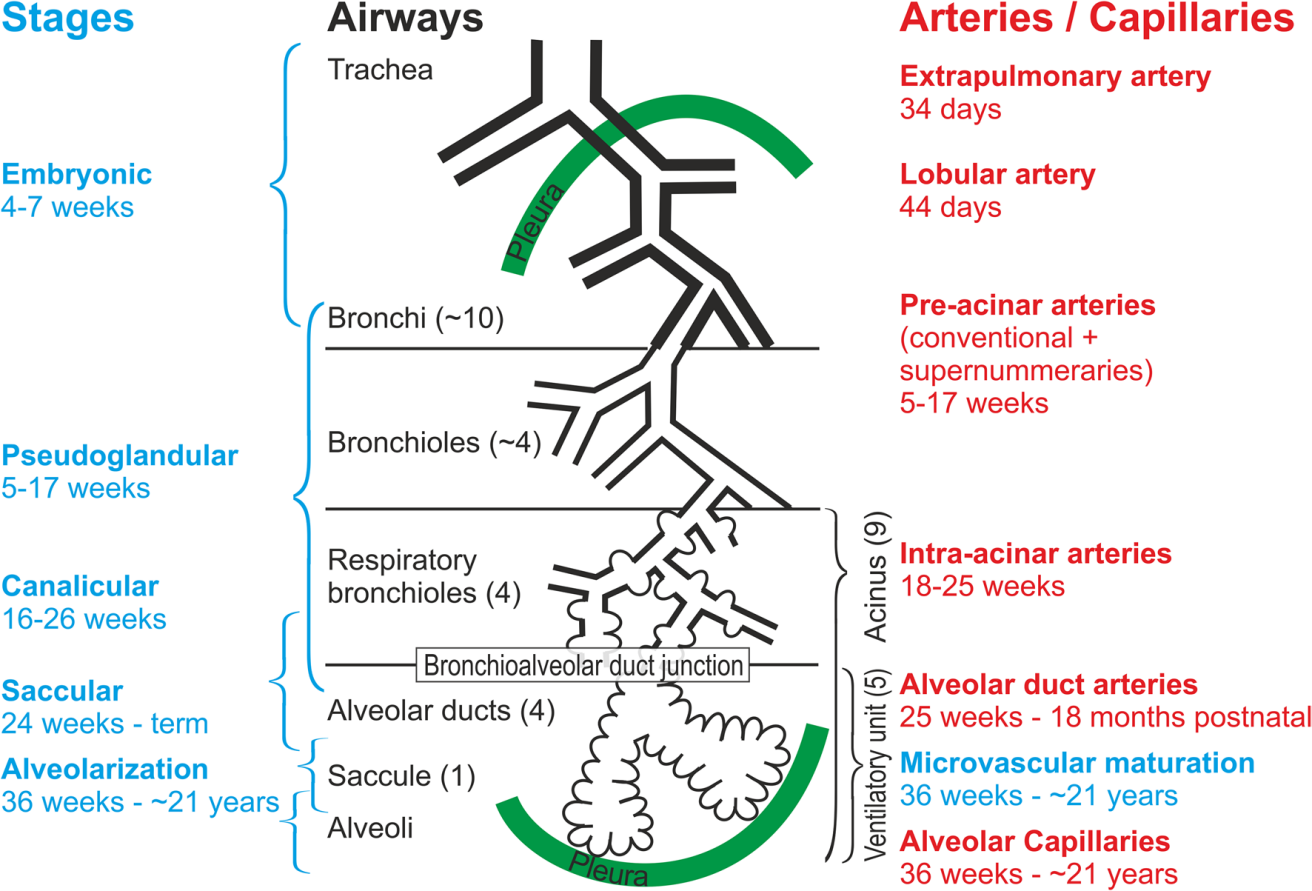
Overview of lung development—development of the airways and blood vessels in humans. Lung development is subdivided into morphologically defined stages (blue). These stages are correlated to the development of the airways (black) and blood vessels (red). On average a human airway ends after 23 generations in an alveolar saccule. Because the shape of the lung is defined by the shape of the thorax and not by the branching pattern of the airways, a range of 18–30 generations has been observed. A capillary plexus surrounding the growing lung buds give rise to the pre-acinar arteries by the mechanism of vasculogenesis. The intra-acinar arteries are formed by angiogenesis. An acinus is defined as a small tree of airways served by a most distal purely conducting airway (terminal bronchiole). A ventilatory unit is defined as a tree of alveolar ducts served by a most distal respiratory bronchiole. In mice and rats the acini and the ventilatory units are the same entity because they do not possess any respiratory bronchioles. Based on Hislop (2005) and adapted from Schittny (2014). By courtesy of Springer Nature Switzerland, Basel

Mammalian lung development starts with the formation of two lung anlagen, one for each lung [for a detailed review about lung development see (Schittny 2017)]. The anlagen are two independent outpouchings of the ventral wall of the primitive foregut (Cardoso and Lu 2006). Both are elongating and start a repetitive circle of growth into the surrounding mesenchyme and branching (branching morphogenesis). The first stage of lung development, the embryonic period (Fig. 1; Table 1), is completed with the formation of the pleura. The latter does not only separate the lungs form the pleural cavities, but also the lung lobes.

**Table 1:** Stages of lung development and their time scale From (Schittny 2017) and by courtesy of Springer Nature Switzerland, Basel

Period	Stage	Duration	Characteristics
Embryonic	Embryonic	Rabbit: n.d.–E18 Sheep: E17–E30 Mouse: E9.5–E12 Rat: E11–E13 Monkey n.d.–E55 Human: E26–E49 (4–7 weeks^a^)	Anlage of the two lungs; organogenesis; formation of major airways and pleura
Fetal	Pseudoglandular	Rabbit: E18–E24 Sheep: E30–E85 Mouse: E12–E16.5 Rat: E13–E18.5 Monkey E55–E85 Human: E35–E119 (5–17 weeks^a^)	Formation of bronchial tree and large parts of prospective respiratory parenchyma; birth of the acinus even if the acinar epithelia are not yet differentiated
	Canalicular	Rabbit: E23–E27 Sheep: E80–E120 Mouse: E16.5–E17.5 Rat: E18.5–E20 Monkey E75–E115 Human: E112–E182 (16–26 weeks^a^)	Formation of the most distal airways leading to completion of branching morphogenesis; first air-blood barrier; appearance of surfactant, acini are detectable due to epithelial differentiation
	Saccular or terminal sac	Rabbit: E27–E30 Sheep: E110–E140 Mouse: E17.5–P4 Rat: E21–P4 Monkey E105–term Human: E168–E266 (24–38 weeks^a^)	Expansion of (future) airspaces
	Alveolarization, classical alveolarization (first phase)	Rabbit: E30–term (E31) Sheep: E120–term (E145)	Formation of secondary septa (septation) resulting in the formation of the alveoli; most of the alveolar septa are still immature and contain a double layered capillary network
Postnatal		Mouse: P4–P21 Rat: P4–P21 Monkey E125 to < P180^b^ Human: E252 (36 weeks^a^ preterm)—3 years	
	Alveolarization, continued alveolarization (second phase)	Rabbit: term (E31)—n.d. Sheep: term (E145)—n.d. Mouse: P14—young adulthood (~ P36) Rat: P14—young adulthood (~ P60) Monkey < P180^b^—young adulthood (7–8 years) Human: 2 years—young adulthood (17–21 years)	Formation of secondary septa (septation), but now lifting off of mature alveolar septa containing a single layered capillary network
	Microvascular maturation	Rabbit: n.d. Sheep: n.d. Mouse: P4—young adulthood (~ P36) Rat: P14—young adulthood (~ P60) Monkey n.d. Human: ~term—~3–21 years (timing uncertain)	Remodeling and maturation of interalveolar septa and of the capillary bed (the double layered capillary network is transformed to a single layered network). In a first approximation it takes place in parallel to alveolarization

The stages are defined mainly by morphological criteria and their beginning and end do not represent sharp borders. In addition, stages are overlapping and regional differences are also common—especially between central and peripheral regions. Furthermore, litter size and nutrition influences the exact timing of development (Miettinen et al. 1997; Bryden et al. 1973; Burri 1999; Ten Have-Opbroek 1991; Schittny et al. 1998, 2008)

*Monkey* Rhesus monkey, *E* embryonic day (days post coitum), *n.d*. not determined, *P* postnatal day

^a^Weeks post coitum

^b^Own unpublished observation

During the next stage, most of the future airways are formed by branching morphogenesis. At this stage the lung looks like a gland which gave this stage its name pseudoglandular stage (Fig. 1; Table 1). The latter is not surprising because branching morphogenesis represents an evolutionary very old mechanism to form branched epithelial tube. This mechanism is also used for the formation of glands (Hannezo et al. 2017), the trachea of insects (Hayashi and Kondo 2018) and others. The epithelial differentiation starts from proximal to distal, forming first the pseudostratified bronchial epithelia. The cuboidal epithelia of the terminal ends of the bronchial tree maintain their undifferentiated state until branching morphogenesis is completed during the canalicular stage. In some species such as human undifferentiated epithelia are still present at these locations during the saccular stage, which may be an indication that branching morphogenesis may continue until the end of the saccular stage (Burri 1985).

During the canalicular stage (Fig. 1; Table 1) the differentiation of the epithelia allows the distinction between conducting and gas-exchanging airways. It permits the detection of the acinus/ventilatory unit for the first time (Boyden 1971), even if most of the acinar airways are already formed during the pseudoglandular stage. Until recently an investigation of the structural development of the acini was not feasible, because the borders between the acini could not be detected in lung sections. It requires 3D-methods which were not easily available (see below).

Epithelial differentiation includes the formation of the bronchioalveolar duct junction. The latter is home of stem cells (Giangreco et al. 2002; Kim et al. 2005) and stays constant throughout live (Barre et al. 2016). Towards the end of the canalicular stage the air-blood barrier matures and a survival of prematurely born babies becomes possible.

90% of the adult gas-exchange surface area will be formed by alveolarization. Pulmonary alveolarization represents a unique mechanism which is fundamentally different from branching morphogenesis. The switching from branching morphogenesis to alveolarization takes place during the saccular stage (Fig. 1; Table 1) (Schittny 2017).

**Fig. 2:**
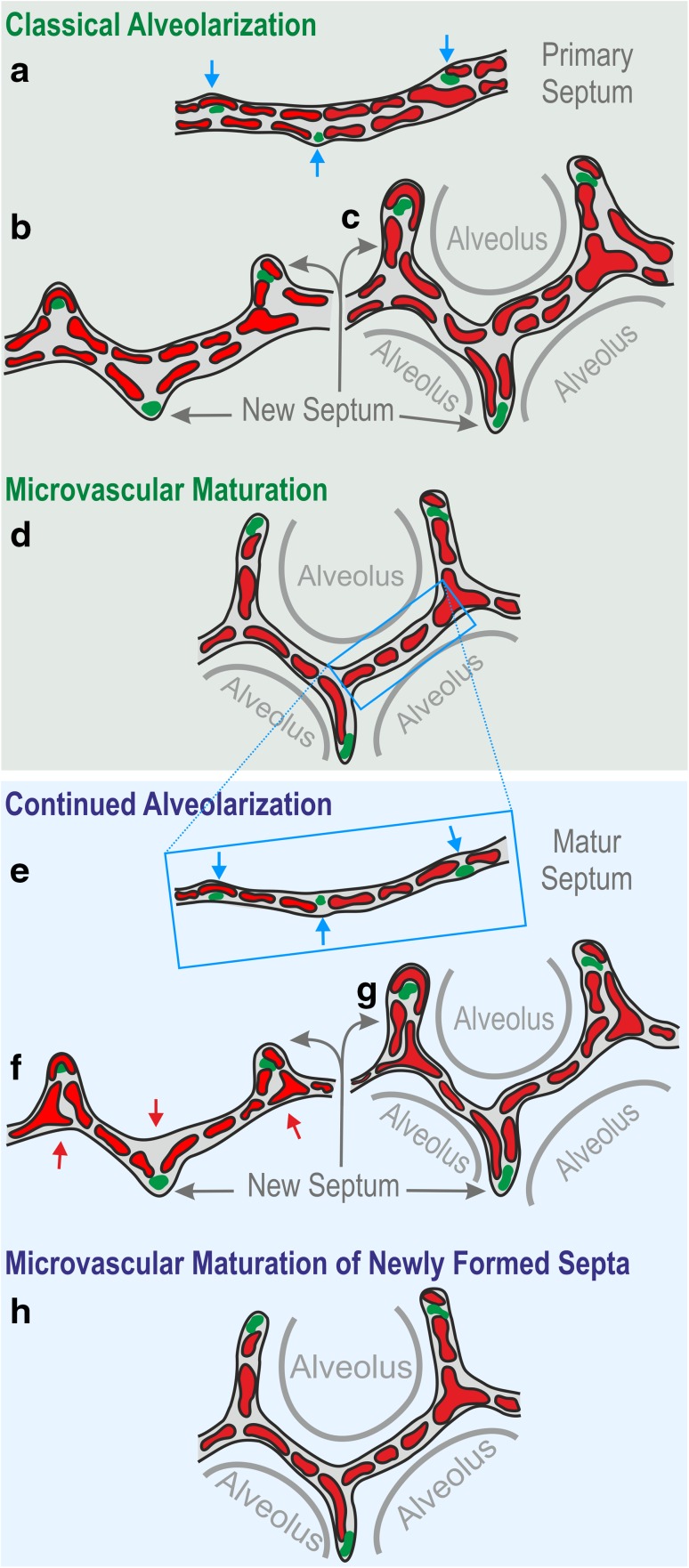
Model of classical and continued alveolarization. Classical alveolarization. **a** During the saccular stage thick interairspace septa were formed due to a condensation of the mesenchyme. These septa are called primary septa and contain a double layered capillary network. At sites where new (secondary) septa will be formed (blue arrow) smooth muscle cells, elastic fibers, and collagen fibrils (green spots) accumulate. **b** The new septa (grey arrows) are formed by an upfolding of one of the sheetlike capillary layers (red). They subdivide preexisting airspaces and by this form the first alveoli. Due to the folding process, the new septa also contain a double layered capillary network (**c**). **d** During microvascular maturation the two capillary layers of the immature septa are reduced to a single layered capillary network. It is believed that this maturation of the alveolar septa increases the efficiency of the gas-exchange. Continued alveolarization. **e** Alveolarization and microvascular maturation takes place more or less in parallel (Fig. 7). Therefore, a significant fraction of the secondary septa is formed rising up form preexisting mature septa which contain only a single layered capillary network. Following the mechanism of continued alveolarization new septa are formed again at sites (blue arrow) where smooth muscle cells, elastic fibers, and collagen fibrils (green spots) accumulate. The capillary layer folds up (red, **d**–**f**), even if the alveolar surface opposing the upfolding is now missing its capillaries (**f**). These “missing capillaries” are immediately formed by angiogenesis (red arrows in **f**). Therefore, regardless when and where new alveolar septa are formed, the sheetlike capillary network folds up resulting in a double layered capillary network in the immature septum (**b, c, f, g**). The septum itself will mature shortly afterwards by a fusion of the two capillary layers into one (**h**). Altered and extended from Burri (1999, 1997), Schittny and Mund (2008, Woods and Schittny (2016) and Schittny (2017), by courtesy of Springer Nature Switzerland, Basel

**Fig. 3:**
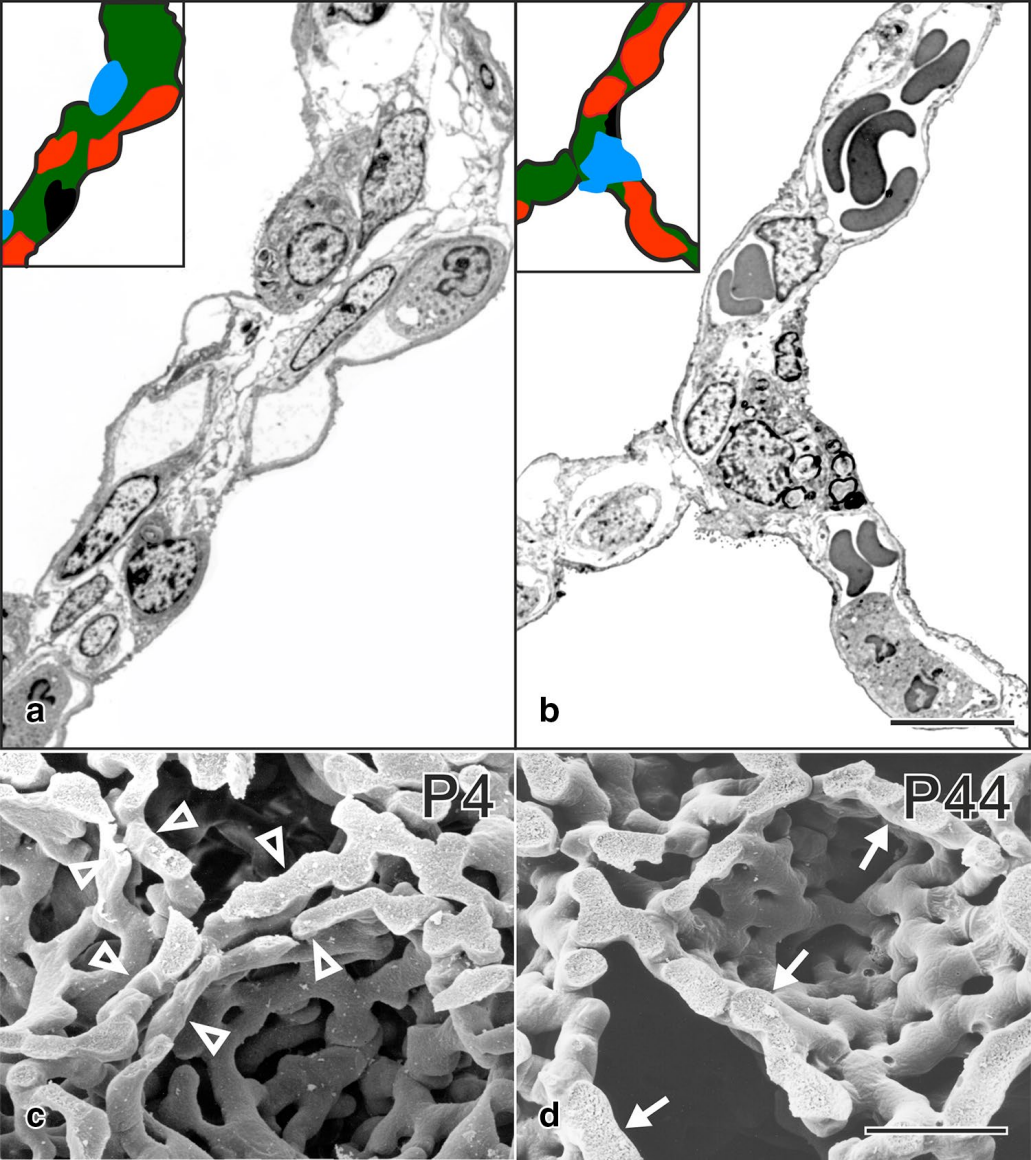
Microvascular maturation/maturation of the alveolar septa. Immature septa contain a double layered capillary network (red) and a central sheet of interstitial tissue (green: **a, c**). To increase the efficiency of the lungs the septa mature. This includes a reduction from a double to a single layered capillary network. The central septum of connective tissue (green) is reduced to a septum interwoven with the capillary network (**b, d**). **a, b** Transmission electron micrographs of human lungs postnatal day 26 (**a**) and adult (**b**). **c, d** Scanning electron micrographs of vascular casts (Mercox^Ⓡ^) of rat lungs at postnatal days 4 (**c**) and 44 (**d**). Bars, **a, b** 10 µm; **c, d** 25 µm. From Schittny (2017) and Woods and Schittny (2016) by courtesy of Springer Nature Switzerland, Basel

Lung development is completed by the phase of alveolarization (Figs. 1, 2; Table 1) and microvascular maturation (Figs. 1, 3; Table 1). During alveolarization preexisting airspaces are subdivided by the formation of new alveolar septa in a repetitive manner. As a result the active surface area for gas-exchange is enlarged in rats by a factor of 18 and the number of alveoli by a factor of 22, but the parenchymal volume (volume of the gas-exchange area) “only” by a factor of 11 (Tschanz et al. 2003, 2014; Burri et al. 1974).

**Fig. 4:**
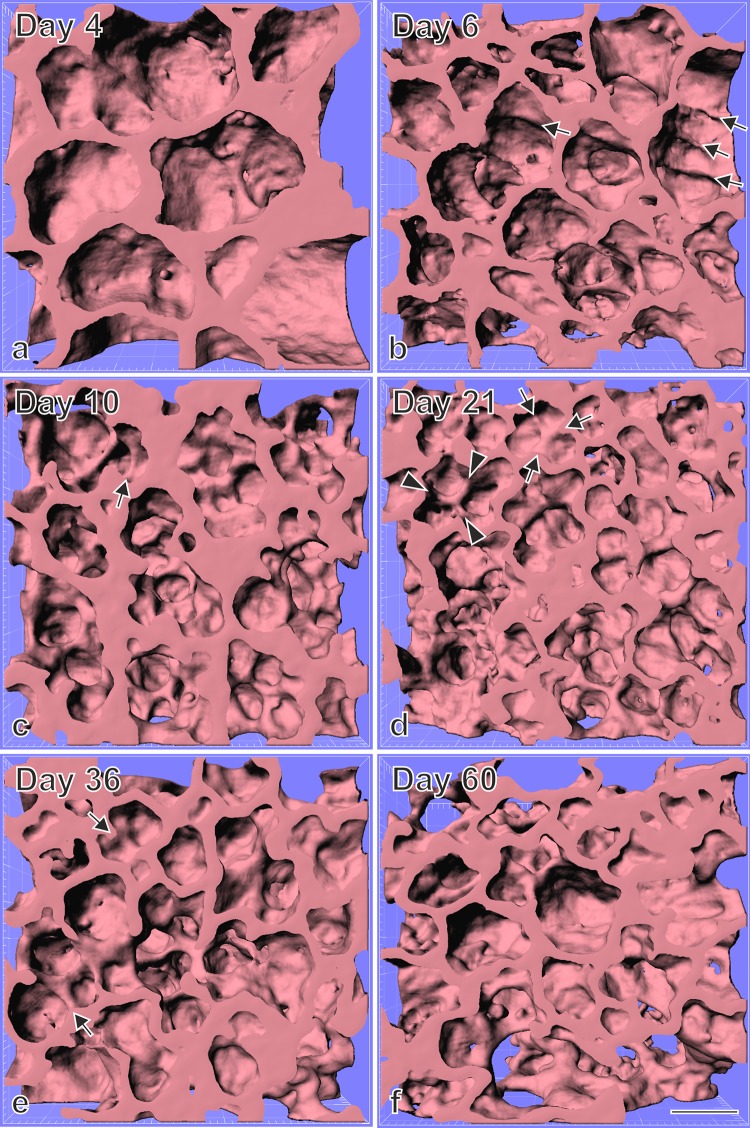
Visualization of alveolarization using synchrotron radiation based X-ray tomographic microscopy of rat lung parenchyma at postnatal days 4–60. Newly forming alveolar septa are recognized by low ridges which are upfolding from existing airspaces. As expected none of these ridges were observed in the larger saccules present during the saccular stage (postnatal day 4, **a**) but many after the start of alveolarization at days 6 and 10 (classical alveolarization, **b, c**, arrows). However, unexpectedly the same structures were observed at days 21 and 36 (**d, e**, arrows) which was taken as an indication that alveolarization continues at least until young adulthood. At day 60 the number of low ridges was below the limit of detection. Synchrotron radiation based X-ray tomographic microscopy was applied for 3D-imaging of lungs embedding for electron microscopy. Arrow head, mature septa; bar, 50 µm. Due to the perspective view the bar is only correct at the surface of the sample. From Mund et al. (2008), by courtesy of Springer Nature Switzerland, Basel

During classical alveolarization, one layer of the double layered capillary network inside the preexisting septa folds up to form a new alveolar septum (Fig. 3). The septa are formed as low ridges which will grow in height to complete the subdivision of the preexisting airspace (Fig. 4). Burri et al. rightly recognized that alveolarization requires a double layered capillary network at the location where the new septa are forming (Burri 1974, 1984; Burri et al. 1974). However, during microvascular maturation the double-layered capillary network of the immature septa fuses to a single layered network resulting in mature septa. It was concluded that after microvascular maturation was completed the formation of new alveoli or alveolar septa, respectively, was not possible anymore (Caduff et al. 1986; Burri 1975).

However, applying the above mentioned new techniques, it was shown for rabbits (Kovar et al. 2002), rhesus monkey (Hyde et al. 2007), rats (Schittny et al. 2008), mice (Mund et al. 2008), and humans (Herring et al. 2014; Narayanan et al. 2012) that alveolarization continues at least as long as the lungs are growing. Furthermore, it was shown that after pneumonectomy a re-growth of the lung including the formation of new alveoli is not only possible in rats, mice, and dogs (Hsia and Johnson 2006; Brown et al. 2001; Brody et al. 1978), but also in humans (Butler et al. 2012). For the details how continued alveolarization, the alveolarization after the maturation of the alveolar microvascular maturation (Fig. 2e–h) was established see below “Change of paradigm for the phase of alveolarization”.

## Overview of modern microscopic and quantitative histological techniques applied for the study of postnatal lung development

### Synchrotron radiation base X-ray tomographic microscopy (SRXTM)

In one aspect lung imaging is nearly like squaring the circle. Very fine structures like the 5–10 µm thick alveolar septa have to be imaged very reliably in a very large volume. Even worse, the third dimension has to be preserved, because it is not possible to recognize the acini in classical 2-dimensional (2D) sections of lung tissue. High resolution X-ray tomography represents the best compromise of resolution and field of view. Magnetic Resonance Imaging (MRI) does not (yet) reach the required resolution and electron microscopical tomography is limited in the field of view. X-ray tomography has the advantage that various methods of sample preparation may be used, as well as live small animals such as mice and juvenile rats (Lovric et al. 2017a). To achieve the required resolution high-resolution bench-top µCT or synchrotron radiation based X-ray tomographic microscopy (SRXTM) was applied. A spatial resolution of 2 µm or less allows not only a detailed recognition and characterization of the conducting airways (Lee et al. 2008), but also a (semi)automated segmentation of acini, followed by their morphological and/or stereological characterization (Vasilescu et al. 2012; Kumar et al. 2013; Haberthur et al. 2013; Xiao et al. 2013, 2016; Kizhakke Puliyakote et al. 2016a). Comparing µCT and SRXTM, SRXTM possesses the advantage of being operated with a ~ 1 billion times brighter and nearly parallel beam instead of the cone beam of the µCT. Furthermore, in SRXTM a highly coherent, monochromatic beam may be used which represents a prerequisite for high quality high-resolution phase contrast imaging (Stampanoni et al. 2014, 2010; Lovric et al. 2016; Vogiatzis Oikonomidis et al. 2017a, b). However, recently phase contrast imaging is getting available for µCTs, too.

In addition to pure imaging, tools for image analysis had to be developed. Due to the large number of tools only some of them could be named in this review, mainly tools which were used for lung development. Due to the structural differences of the conducting and gas-exchanging airways, different tool boxes had to be used. Lee et al. analyzed the conducting airways throughout rat lung development and determined segment lengths and diameters, as well as the branching angles (Lee et al. 2011). As global measures of parenchymal properties airspace size (thickness map) and the curvature of the alveolar surface where estimated in 3D in postmortem mice (Lovric et al. 2013, 2017a, b). In addition, ventilation and perfusion distributions, as well as ventilation induced lung injury present another field of the application of SRXTM (Bayat et al. 2001, 2013; Ito et al. 2013; Broche et al. 2017). The characterization of the size, number, surface area, and skeleton of the acini in adults and during development should also be named (Haberthur et al. 2010; Lovric et al. 2013, 2017a, b; Kizhakke Puliyakote et al. 2016a, b; Scott et al. 2015; Vasilescu et al. 2013; Xiao et al. 2016). At last but not at least SRXTM is also used to characterize the alveolar microvasculatur during development, repair, and disease (Ackermann et al. 2014; Schittny et al. 2008). The latter two, the development of the acini and the microvascular during alveolarization will be discussed in more detail (see below).

### Design-based stereological

To understand the structural development of the lung including its functional implication an unbiased method is needed for an efficient quantification of number, length, surface area, and volume. In addition, the method has to be free of geometric assumptions. Furthermore, accuracy is more important than precision, because a biased or inaccurate design cannot be corrected by increasing the number of measurements. These principles have to be applied to all steps of the process, e.g. tissue fixation, embedding, sampling, and analysis. In the early 1960s Weibel at al. developed such a method, the design-based stereology, to investigate mature, developing, and diseased lungs (Weibel 1962, 1963; Weibel and Gomez 1962). It is even possible to estimate physiological parameters, e.g. the pulmonary diffusion capacity (Siegwart et al. 1971; Geelhaar and Weibel 1971; Burri and Weibel 1971; Weibel 1970; Weibel and Kistler 1965). Stereology represents the gold standard for any quantitative characterization of the lung.

Postnatal rat lung development was first described quantitatively by Burri et al. in the early 1970s (Burri 1974, 1975; Kauffman et al. 1974; Burri et al. 1974). They characterized the development of the parenchymal volume, alveolar surface area, and the number of various cell types. However, at that time they were not able to determine the number of alveoli design-based and unbiased.

The situation changed when Hyde et al. (2004) developed a design based method to count the number of alveoli and when the group of the author used the length of the free septal edge to follow alveolarization (Schittny et al. 2008; Mund et al. 2008). Both methods were developed based on two different points of view: alveolarization, the formation of new alveoli, versus septation, the formation of new alveolar septa.

As typical for any unbiased counting which is independent of the size and shape of the element of interest, Hyde at al. (2004) applied the so called disector. A disector consists of two consecutive sections with a constant and known distance between them. A structural element will be counted when it is present in one section but not in the other one. The number of alveoli was estimated by counting the alveolar openings at the level of the free septal edges. Mathematically, the Euler characteristic of the network of alveolar entrance rings was determined which represents the number of alveolar entrances. In addition to the number of alveoli, the number weighted mean alveolar volume could be determined after estimating the total alveolar volume (Ochs et al. 2004; Muhlfeld et al. 2015; Hsia et al. 2010; Hyde et al. 2004). As a first application of this method, the number of alveoli was counted in an adult lung by Ochs et al. (2004).

The formation of new septa could be determined by an estimation of the total length of the free septal edge and a comparison between different days of lung development. In a counting frame on lung sections the number of septal tips was counted and the length density and total length were calculated. Based on the total length of the free septal edge and the parenchymal volume a distinction between increase of septal length due to the growth of the lungs during development and due to the formation of new septa was done in addition. As a first approximation, the lung parenchyma grows isometric, meaning geometric similar. Based on this approximation the percentage of newly forming septa (anlage of septa) could be calculated (Schittny et al. 2008; Mund et al. 2008).

### Hyperpolarized gas magnetic resonance imaging

^3^He and/or ^129^Xe hyperpolarized Magnetic Resonance Imaging (MRI) allows pulmonary diffusion measurement in vivo. The combination of diffusion measurements with variable diffusion times, diffusion-sensitizing gradients, and modeling of gas diffusion in lung airspaces permits the estimation of quantitative structural information at alveolar level. This approach was named in vivo lung morphometry and allows a structural characterization of a living lung (Yablonskiy et al. 2017). It was utilized to show that juvenile humans still form new alveoli, even if the alveolar septa are believed to be mature at an age 2–3 years (Herring et al. 2014; Narayanan et al. 2012).

## Change of paradigm for the phase of alveolarization

The requirement of a double layered capillary network for the formation of new alveolar septa was rightly recognized during the early 1970s (Burri 1974, 1984; Burri et al. 1974). As a consequence it was proposed that after microvascular maturation (day 21 in mice and rats; second to third year of life in humans) no new alveoli would be formed anymore (Caduff et al. 1986; Burri 1975). This hypothesis excluded any kind of structural lung repair. However, about a decade later various evidence for some kind of late alveolarization accumulated. E.g., after pneumonectomy a re-growth of the lung parenchyma was reported not only for animals (Hsia et al. 1994; Cagle and Thurlbeck 1988), but later also for humans (Butler et al. 2012). Furthermore, a short neonatal dexamethasone treatment of rats reduced alveolarization and induced premature microvascular maturation (Roth-Kleiner et al. 2005; Luyet et al. 2002). In juvenile rats late alveolarization was observed which rescued the phenotype (Tschanz et al. 2003; Schwyter et al. 2003). A similar phenomenon was observed after a longer dexamethasone treatment followed by a treatment with retinoic acid (Massaro and Massaro 2000) and after starvation and re-feeding (Coxson et al. 2004; Kalenga et al. 1995a, b). As further evidence for late alveolarization, low ridges, which are indicative for the formation of new alveolar septa, where observed in rats lungs at days 21–36 in scanning electron micrographs of lung parenchyma (original data not shown, SRXTM, which were obtained later are shown in Fig. 4). At these time points most of the alveolar septa are matured (Schittny et al. 2008). Therefore, there was overwhelming evidence that alveolarization does not stop after microvascular maturation was completed.

**Fig. 5:**
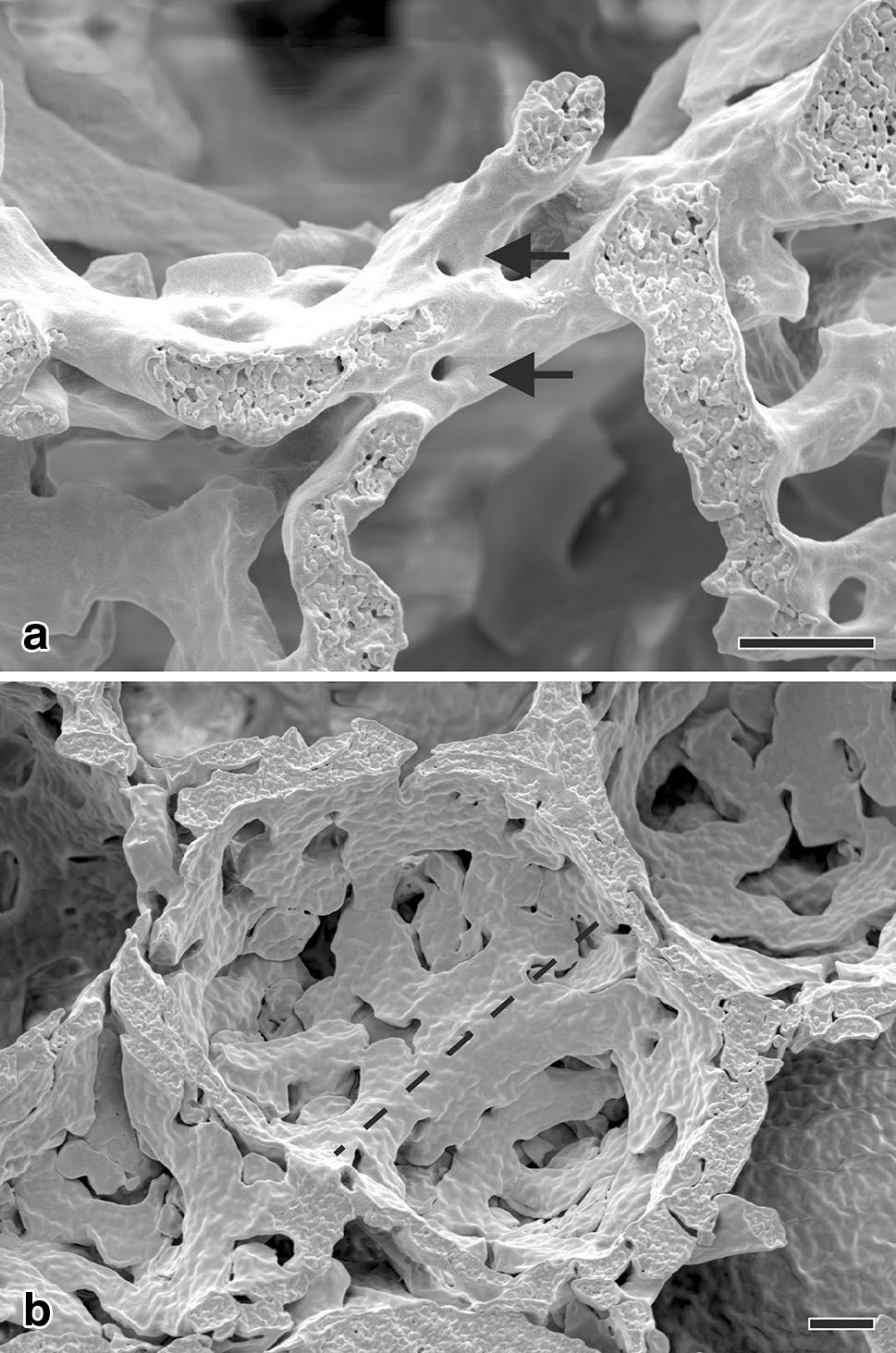
Scanning electron microscopy of vascular casts (Mercox^®^) of 21-day-old rat lungs. To decipher the contribution of the capillary network to continued alveolarization, we were searching for tissue posts in the sheetlike capillary network which were orientated in the plane of the capillary layer (**a**). We took it as a morphological indication of a capillary splitting/duplication. In addition, we searched for upfoldings of the capillary network as an indication of the formation of new alveolar septa (**b**). However, we did not find any location, which showed both events in the same image—which would have been necessary to understand the mechanism of continued alveolarization. Therefore, scanning electron microscopy of vascular casts (Mercox^®^) did not enable us to postulate a hypothesis how new alveolar septa may be formed after the maturation of the microvasculature of the alveolar septa. Bar 20 µm

**Fig. 6:**
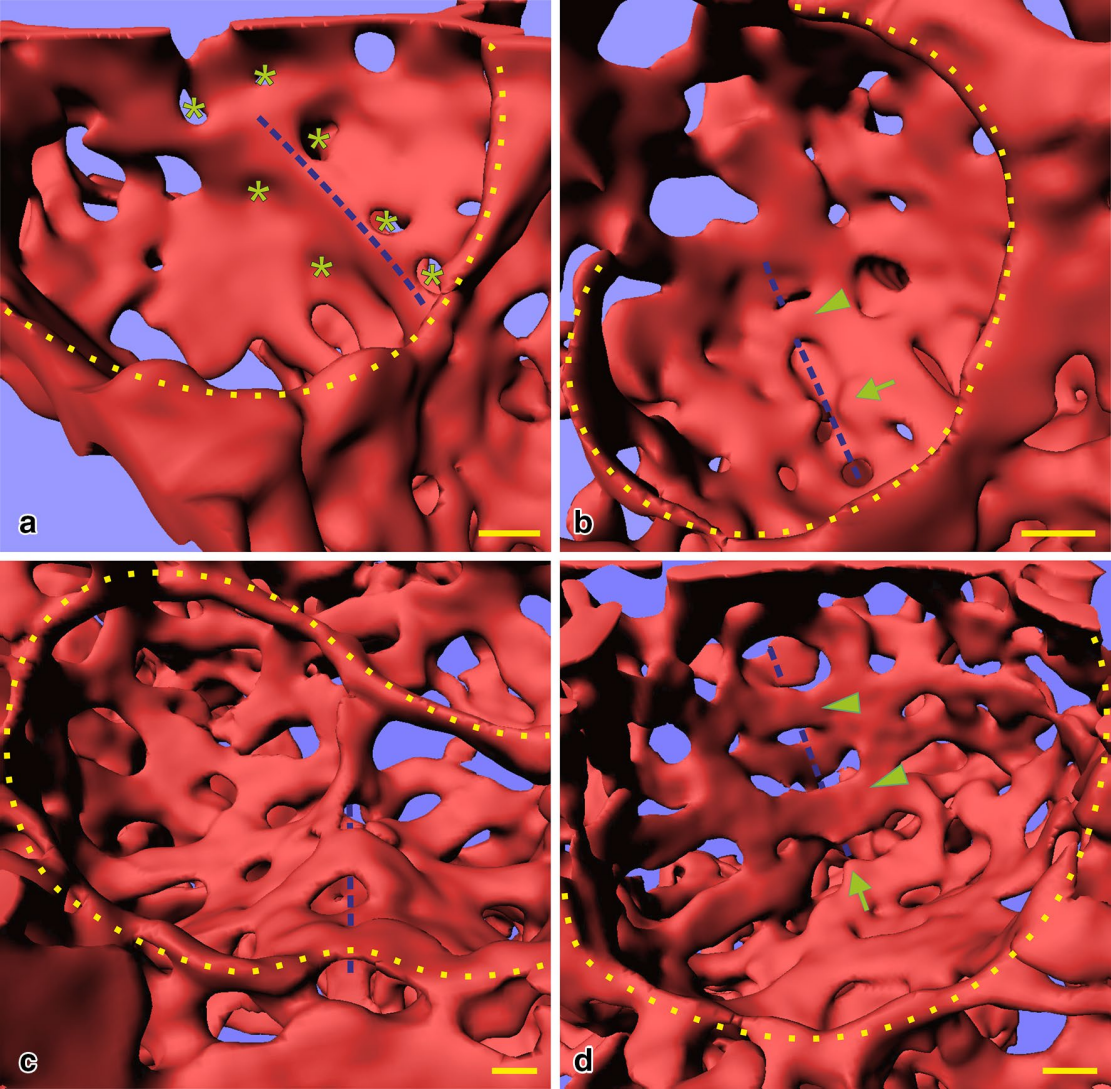
Duplication of the sheetlike capillary layers during continued alveolarization. Visualizations of vascular casts (Mercox^®^) of 21 days old rat lungs after synchrotron radiation based X-ray tomographic microscopy. The lumen of the capillaries is shown as a surface rendering. This surface is identical with the inner surface of the capillaries. Inside the cavity of two alveoli (**a, c**) an upfolding of the sheet-like capillary network was detected (blue dashed line). As stated in the legend of Fig. 5 the upfoldings are an indication of the formation of new alveolar septa. In opposite to the scanning electron microscopical samples (Fig. 5) the tomographic dataset permitted to analyze the backside or the same septum (**b, d**). At the basis of the newly forming septum a local duplication of the existing capillary network was detected (blue dashed line in **b, d**). While parts of the duplication are already formed (arrowhead), the remaining duplication is just forming—most likely by sprouting angiogenesis (arrow). The asterisks label tissue pillars in the capillary layer which are indicative for intussusceptive angiogenesis (Caduff et al. 1986) at the basis of the new septum. The yellow dotted line labels the entrances of the alveoli. Bar 10 µm (the magnification varies inside the image due to the foreshortened view). **a, b** From (Schittny et al. 2008) and by courtesy of Springer Nature Switzerland, Basel

However, what could be the mechanism? At the turn of the century, the author had his own group in the laboratory of Peter Burri at the Institute of Anatomy at the University of Bern. It was obvious to use scanning electron microscopy to study vascular casts of the alveolar microvasculature, because this method was originally used to describe the process of microvascular maturation itself (Caduff et al. 1986; Burri 1975). We had the following hypothesis: the single layered capillary layer of a mature septum will be split by intussusceptive angiogenesis and one layer will fold up to form the new septum while the other remains in the preexisting septum. As evidence of this kind of angiogenesis we searched for tissue pillars in vascular cast (Mercox^®^) of the alveolar microvasculature during lung development (Fig. 5a). In addition, we searched for upfoldings of one of the two capillary layers as an evidence of the formation of new alveolar septa (Fig. 5b). However, we never observed any combination of the two events which would have been an indication for the mechanism of late alveolarization. After spending half a year at the scanning electron microscope we gave up. Later, we got into contact to the group running the X-ray tomographic microscope at the Swiss Light Source (Stampanoni et al. 2002). To my best knowledge at this time this microscope represented the “µCT” with one of the highest resolutions. It was much higher than commercial µCT. We took our samples prepared for the fortuneless scanning electron microscopical study, scanned them and did 3D-visualization. Because now we were able to look form every possible angle to any sub-volume, it took only one day to understand the contribution of the microvasculature to the formation of new alveolar septa from preexisting mature septa (Fig. 6). The single layered capillary network starts to fold up at the location where the new septum is forming. The required second capillary layer is immediately formed by angiogenesis, most likely by sprouting and not by intussusceptive angiogenesis (Figs. 2, 6) (Schittny et al. 2008). To emphasize that the first phase of alveolarization represents the classically described mechanism this phase is called classical alveolarization. The second phase represents a continuation of the first one with the only difference that one “missing” capillary layer is formed instantly. Therefore, it is called continued alveolarization (Tschanz et al. 2014; Schittny et al. 2008).

**Fig. 7:**
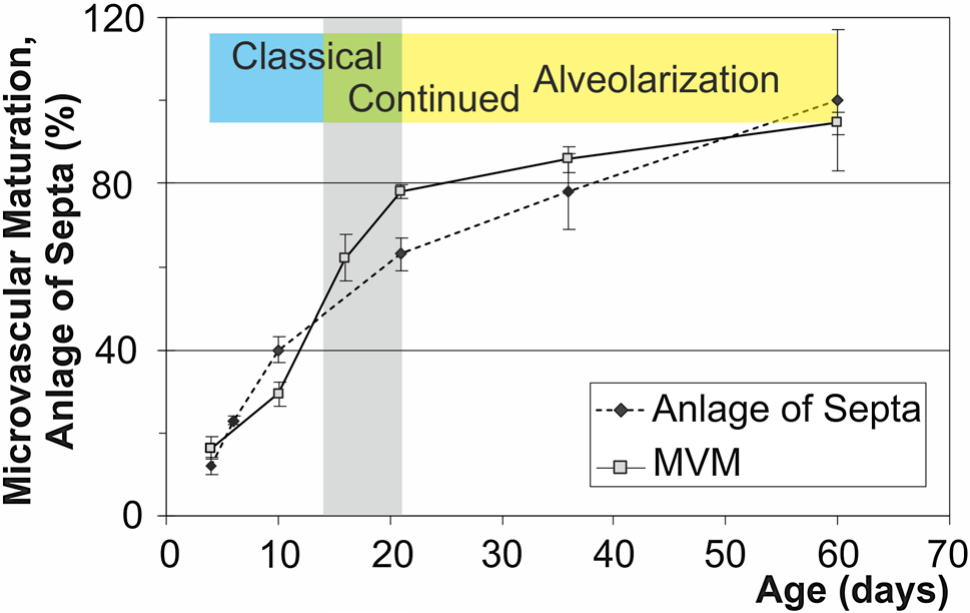
Comparison of microvascular maturation and alveolarization in rat lungs. New alveolar septa are formed steadily. Their formation is faster at the beginning of alveolarization and slows down later (dotted line). Originally microvascular maturation was defined based on morphological observations. The bulk of it was believed to take place between postnatal days 14–21 (period labeled in grey), (Burri 1975). A stereological estimation of microvascular maturation revealed that this process starts in parallel to alveolarization and levels off at 95% of maturation when alveolarization ceases (solid line). The anlage of new septa was calculated based on the estimation of the length of free septal edge. (Schittny et al. 2008). Microvascular maturation was calculated based on the estimation of the alveolar surface area overlaying a single (mature) or double layered (immature) capillary network (Roth-Kleiner et al. 2005). For alveolarization day 60 was defined as 100%. Data from Roth-Kleiner et al. (2005, Schittny et al. (2008) and Schittny (2017). Altered from Schittny (2017) and by courtesy of Springer Nature Switzerland, Basel

To understand the premature maturation of the alveolar septa after a dexamethasone treatment of neonatal rats, microvascular maturation was characterized stereologically throughout lung development. It turned out, that microvascular maturation represents not just a short phase following alveolarization as originally described (Caduff et al. 1986), but takes place roughly in parallel to classical and continued alveolarization. While in rats the formation of alveoli shows a biphasic pattern (Tschanz et al. 2014), microvascular maturation exhibits a sigmoidal behavior (Fig. 7) (Roth-Kleiner et al. 2005).

## Development of the pulmonary acini/ventilatory units

The acinus is defined as the small tree of airways which is served by the most distal purely conducting airway, the terminal bronchiole. In lungs possessing respiratory bronchioles (e.g., humans, rhesus monkeys, dogs, and cats) (Harkema et al. 2018) the acinus starts with few generations of respiratory bronchioles. Proximal of the bronchioalveolar duct junction (BADJ) the ventilatory unit (Storey and Staub 1962) starts. It consist of a small tree of alveolar ducts possessing sacculi at their terminal ends (Schittny 2017). In animals such as mice, rats, and the hamster, which do not possess respiratory bronchioles, the acini and the ventilatory units represent the same structure (Tyler 1983).

**Fig. 8:**
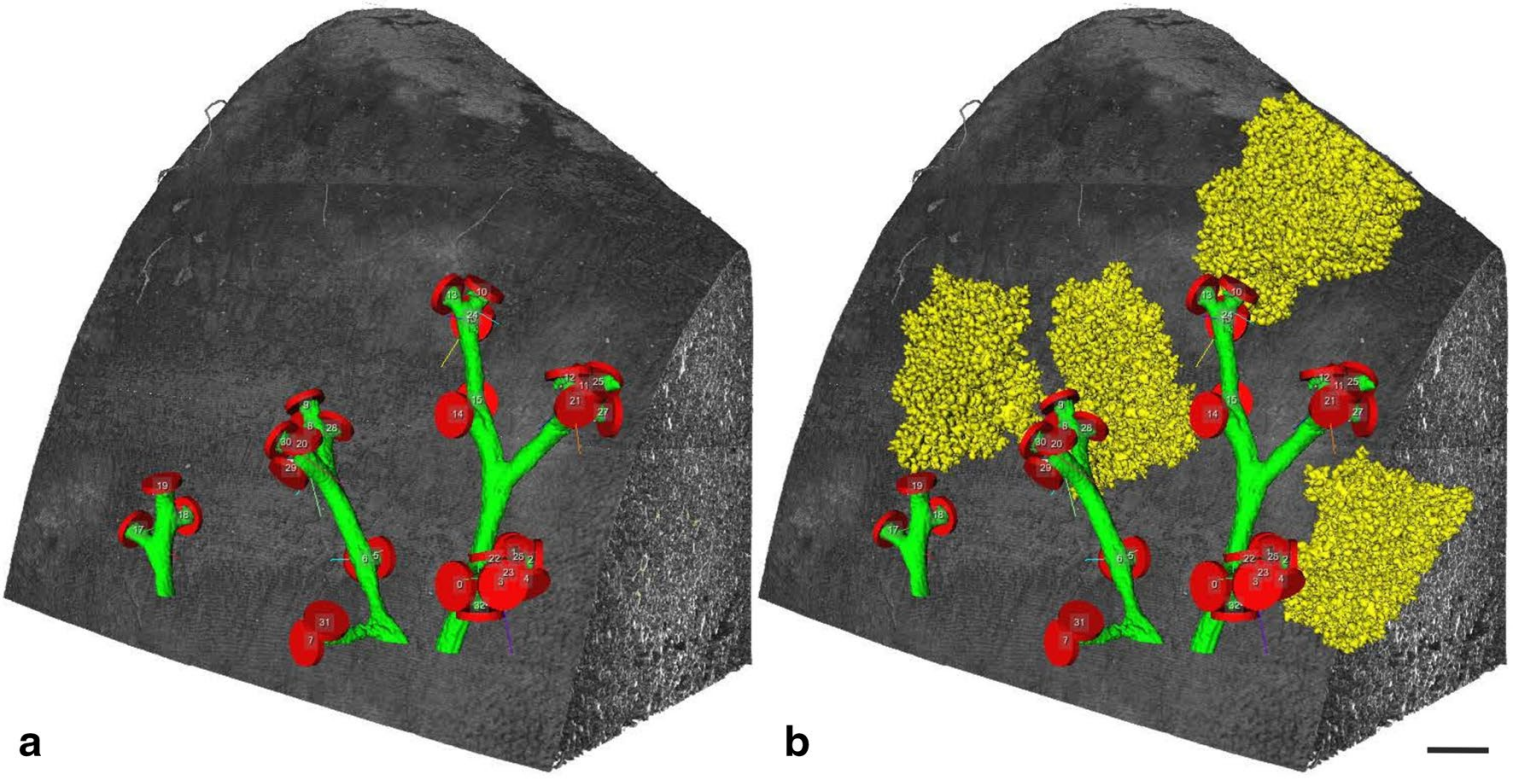
3D-visualization of acini branching off of distal conducting airways in a rat lung. Surface renderings of individual acini are shown in yellow. They are branching off of distal conducting airways (green) at the bronchioalveolar duct junction (labeled by red segmentation stoppers). The lung tissue is shown in shades of grey. **a** Conducting airways closed by segmentation stoppers at the acinar entrance. **b** Four non-neighboring acini are shown in addition of the structures shown in **a**. Bar 0.5 mm. From Haberthur et al. (2013), by courtesy of Springer Nature Switzerland, Basel

Acini are not detectible on lung section, because no marker exists to detect the border between two acini. Therefore, several 3D methods were used to study the structure and development of pulmonary acini. Yeh et al. (1979) and Rodriguez et al. (1987) used casting methods. 3D-reconstructions of individual acini were performed by Mercer and Crapo (1987) using serial lung sections. Wulfsohn et al. (2010) developed a technique based on a disector of five consecutive sections to estimate the number of acini/ventilatory units. Unfortunately, all of these techniques were very labor intensive and, therefore, not suitable to follow the development of the acini during the phases of alveolarization. Several µCT or SRXTM based approaches were used to visualize pulmonary acini (Haberthur et al. 2013; McDonough et al. 2011; Sera et al. 2003, 2005, 2013; Vasilescu et al. 2012) like the ones shown in Fig. 8.

**Fig. 9:**
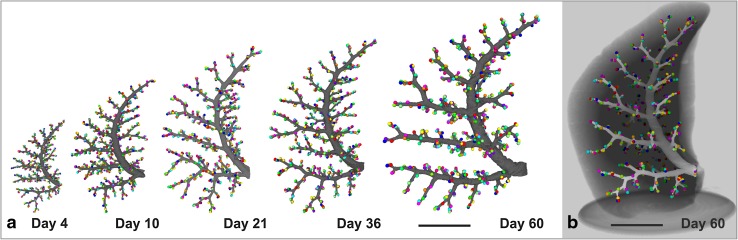
Development of the bronchial tree of the conducting airways of the right middle rat lung lobe. 3D-surface renderings of the outer surface of the conduction airways are shown in grey. The colored balls represent a segmentation stopper placed at entrance of every acinus. A large similarity of the structure of the conducting airways was detected at days 4, 10, 21, 36 and 60 and between different individuals (**a**). **b** The bronchial tree embedded in the surrounding lung parenchyma at day 60. Bar 5 mm. Altered from Barre et al. (2016), by courtesy of Springer Nature Switzerland, Basel

Based on SRXTM images at a resolution of ~ 1 µm and on Wulfsohn et al. (2010) and Barre et al. (2014) developed a protocol for the fast and easy recognition of the BADJ and for the counting of the number of rat acini throughout lung development (Barre et al. 2016). Surprisingly, the number of acini did not change during lung development. Four days old rats possessed the same number of acini as adults, even if the lung volume increased by a factor of ~ 11 during this time. It is obvious that this large increase must have a significant influence on acinar ventilation and particle deposition. As a side effect of the counting the conducting airways and the acinar entrances of one rat lung lobe were visualized in 3D throughout lung development (Fig. 9). A striking similarity was observed between the bronchial trees obtained at different days and between individual animals (each day shown represents a different animal; Fig. 9).

The very large increase of the volume of the acini raised the question if new structural elements are necessary to keep the acinar airways space filling. We tested it by calculating the fractal dimension of individual acini throughout lung development. We observed that the fractal dimension was similar at days 4 and 10, increased at day 36, and even more at day 60. This result predicts that additional still unknown structures are formed and/or that the differential growth of the airways causes an increase of acinar complexity (Vogiatzis Oikonomidis 2018).

## Conclusion

Pushing for the development of new methods opened up new opportunities to refine our view of postnatal lung development. Clinically these refinements are very important because it shows that a cure of structural lung diseases may be possible—or differently expressed, lung regeneration takes place, even if currently no clue exists how to foster the regeneration. Furthermore, structural details obtained at high resolution in 3D are very important for our understanding of ventilation and particle deposition. E.g., they are important for simulations of acinar airflow and particle deposition (Sznitman et al. 2010; Henry et al. 2012; Tsuda et al. 2008; Hofemeier et al. 2018) or for the correlation between the amount of the deposition of inhaled particles in rat lungs and the size of the airspaces (Semmler-Behnke et al. 2012; Kreyling et al. 2018). Therefore, the application of modern cutting edge imaging and quantitative histological techniques advanced our anatomical and physiological knowledge significantly. However, the development of new techniques is getting more and more sophisticated. Therefore, it is typically done in teams of biologist, biomedical engineers, physicists, and physicians. On one hand being a member of such a team is very exciting and stimulating. On the other hand demanding, because every member of the team has to be able to understand the others language and way of thinking.

## Electronic supplementary material

Below is the link to the electronic supplementary material.

Flight into a rat acinus. The flight starts by entering a transitional bronchiole. Domes of Club cell are visible on the surface of the bronchiole. Turning left an alveolar duct is entered. Various alveoli and the entrance of few alveolar ducts are visible. Shortly before the end of first alveolar duct, the fight turns left again and flies down another alveolar duct. After a short distance it ends in front of an alveolus which is subdivided by a low ridge representing a still forming new alveolar septum. Rat lung at postnatal day 36. Surface rendering of a sample scanned by SRXTM for Schittny et al. (Schittny et al. 2008) using the software Imaris 4.1 (Bitplane, Zürich, Switzerland). Because the magnification is changing during the flight a scale bar could not be easily shown. However, the entrance of the bronchiole has a diameter of ~100 µm (WMV 4622 KB)

## Abbreviations

2D: Two-dimensional
3D: Three-dimensional
BADJ: Bronchioalveolar duct junction
MRI: Magnetic resonance imaging
SRXTM: Synchrotron radiation based X-ray tomographic microscopy
